# Retraction: Development and Validation of a Microarray for the Investigation of the CAZymes Encoded by the Human Gut Microbiome

**DOI:** 10.1371/journal.pone.0312333

**Published:** 2024-10-14

**Authors:** The PLOS ONE Editors

The *PLOS ONE* Editors retract this article [1, 2] due to concerns about compliance with the PLOS Human Subjects Research policy.

Specifically, the study reports the use of fecal samples from lean women obtained during a previous study [3, retracted in 4]. However, as indicated in [4], the article reporting the original study was retracted because, “the authors did not confirm approval from an appropriate ethics approval to recruit healthy donors to provide fecal samples for research.” Furthermore, the *PLOS ONE* article [1] does not contain sufficient information to determine when the sample collection for this study took place.

In addition, PLOS identified potential competing interests between the ethics committee of the Institut Fédératif de Recherche (IFR) 48 that granted the ethics approval document #10–002 cited in this article and one or more of the article’s authors.

A representative of the Aix-Marseille Université Ethics Committee stated that the institute disagrees with the retraction decision, and that their investigation has not determined that the article violates French regulations or international ethics rules. They stated that the stool samples collected in this study are considered human waste, and that the study did not require ethics approval from a Comité de Protection des Personnes according to French law.

BH agreed with the retraction. FA and MM did not agree with the retraction; MM stands by the article’s findings and FA apologizes for the issues with the article. AEK, QL, BV, and DR either did not respond directly or could not be reached.

Note: The ethics approval number (10–002) cited in this article’s ethics statement is for a protocol titled, "Metagenomic study of the digestive microbiome". The approval was issued on 11 January 2010 by the ethics committee of IFR48, and has also been cited as supporting the studies reported in [5–10].
